# An improved method for phasing crystal structures with low non-crystallographic symmetry using cryo-electron microscopy data

**DOI:** 10.1007/s13238-015-0219-4

**Published:** 2015-10-27

**Authors:** Jia Wang, Weiguang Wang, Wen Song, Zhifu Han, Heqiao Zhang, Jijie Chai, Hongwei Wang, Jiawei Wang

**Affiliations:** State Key Laboratory of Membrane Biology, School of Life Sciences, Tsinghua University, Beijing, 100084 China; Center for Structural Biology, School of Life Sciences, Tsinghua University, Beijing, 100084 China; Tsinghua-Peking Center for Life Sciences, School of Life Sciences, Tsinghua University, Beijing, 100084 China; Ministry of Education Key Laboratory of Protein Science, School of Life Sciences, Tsinghua University, Beijing, 100084 China

**Dear Editor**,

Cryo-electron microscopy (cryo-EM) and macromolecular crystallography (MX) both allow the creation of three-dimensional images of biological macromolecules. EM data usually provide access to three-dimensional reconstructions of large biological complexes at low resolution, whereas MX usually delivers atomic or quasi-atomic resolution structures (1–3 Å). Two limiting factors of MX, which are not encountered in EM, are the production of high diffraction-quality crystals and the “phase problem”. Therefore, these two methods can be utilized in combination to determine the structure of biological macromolecules, especially of very large and complicated assemblies (Dodson, 2001; Navaza, 2008; Stuart and Abrescia, 2013; Xiong, 2008).

Low-resolution EM reconstructions were previously used to solve high-resolution MX structures by the molecular replacement (MR) method (Rossmann, 1972). The process is divided into two consecutive steps (Fig. 1). In the first step, an EM reconstruction model is placed at the center of a P1 cell with ‘crystal’ unit cell dimensions at least twice the model diameter. The structure factors can be generated by the inverse Fourier transform. Molecular replacement is then performed to match observed crystal diffraction intensities to those predicted by a suitable EM model correctly oriented and positioned within the unit cell (Dodson, 2001). Sufficient resolution overlapping between the EM reconstruction and MX data is the most critical parameter for the success of this step (Jenni and Ban, 2009). In the second step, after the search EM map has been positioned, EM-model based phases are calculated up to the EM reconstruction resolution. Phase extension to the higher resolution provided by the X-ray diffraction data was usually achieved by iterated density-modification procedures comprising solvent flattening, histogram matching, and especially non-crystallographic symmetry (NCS) map averaging. This strategy has been successfully exploited in the determination of structures with a high degree of internal symmetry, e.g. icosahedral viruses (Stuart and Abrescia, 2013), fungal fatty acid synthase (FAS) particle (Jenni and Ban, 2009; Xiong, 2008) with 32-fold symmetry, major vault protein (cpMVP vaults) including concentric 24- and 48-fold rotational symmetry (Anderson et al., 2007), CaDHQ with 12 proper NCS operators and one improper NCS operator (Trapani et al., 2010), bacteriophage φ6 major capsid protein with five-fold NCS (Nemecek et al., 2013), a bacteriophage capsid protein P2 with six-fold NCS (Abrescia et al., 2011), etc. which often results in a good-quality electron density map for interpretation even at the resolution of around 3 Å, due to the power of the NCS averaging technique. To our knowledge, this strategy has never been used for two NCS copies or less in the asymmetric unit (ASU).

**Figure 1:**
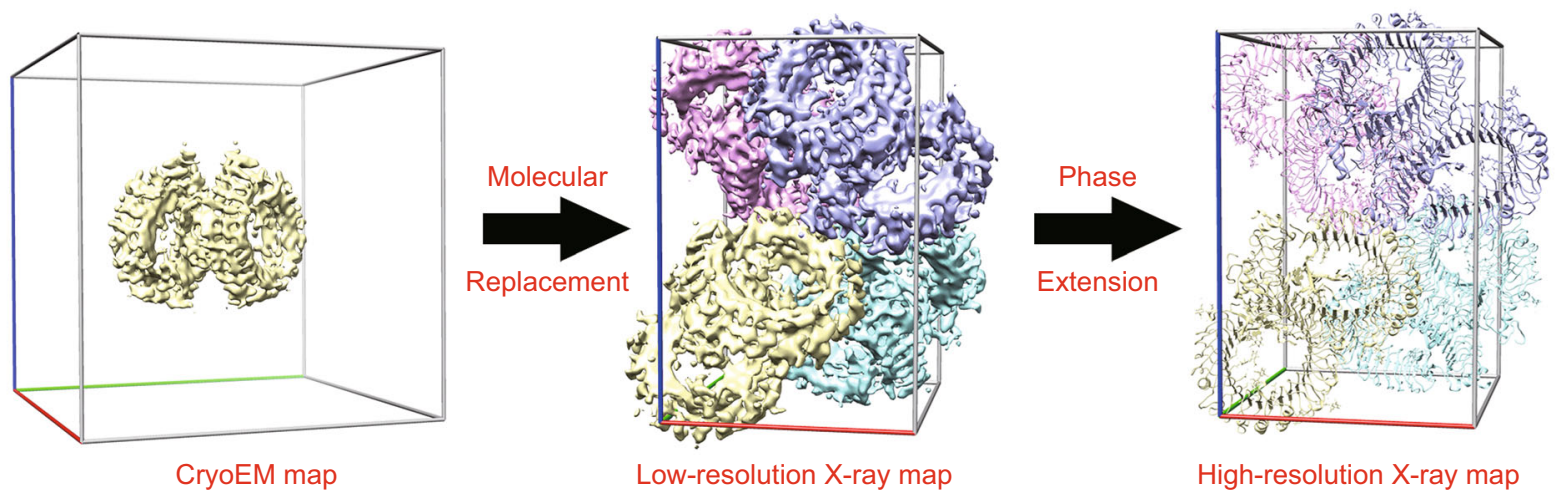
**Schematic representation of the workflow combining EM and MX techniques**. First, EM reconstruction models are placed at the center of a P1 cell with ‘crystal’ axes at least twice the model diameter. The structure factors are generated from inverse Fourier transform. Molecular replacement is then performed to match observed crystal diffraction intensities to those predicted by a suitable EM model correctly oriented and positioned within the crystal unit cell. Second, once the search EM map has been positioned, EM-based phases can be calculated up to the EM reconstruction resolution. Phase-extension procedures are then needed to solve the structure at the resolution provided by the X-ray diffraction data

Recently we applied the EM/MR approach to solve the crystal structure of toll-like receptor 13 (TLR13) with only two TLR13 molecules in the asymmetric unit of the cell. This work demonstrates that the phase extension method described in this study can extend the EM-based phases to higher resolution even without the usual high number of NCS copies.

Toll-like receptors (TLRs) have crucial roles in innate immunity by functioning as pattern recognition receptors. TLR13 recognizes a stretch of conserved nucleotides from bacterial 23S ribosomal RNA with stringent specificity to trigger an immune response (Song et al., 2015). We crystallized the complex of TLR13 ecto-LRR domain with a 13-nucleotide RNA oligomer and collected a native X-ray diffraction dataset of 2.3 Å (Table S1). However, the traditional phasing methods in X-ray crystallography, e.g. molecular replacement with homologue structures, isomorphous replacement with heavy atom derivatives, or anomalous dispersion with selenium methionine substitution, all failed for phasing the TLR13 structure.

Advances in electron microscopy (Kuhlbrandt, 2014) may pave the way for the routine use of low-resolution three-dimensional reconstructions as MR models to phase X-ray data (Stuart and Abrescia, 2013). To solve the phase problem in the TLR13 solution by a cryo-EM map, a single-particle cryo-EM reconstruction at 4.87 Å of TLR13 with a 25-nucleotide RNA oligomer was obtained (Table S2). In the cryo-EM map, two copies of TLR13 molecules formed an “M-shaped” dimer (Fig. 1), which is supposed to be the effective assembly for recognition of single-strand RNA released from the internalized microbes for activation.

Although the correct position of the TLR13 dimer has been identified in the crystallographic data as judged by the high value of Z-score in PHASER (McCoy et al., 2007), and phases were calculated up to 4.87 Å resolution, the low number of NCS copies of TLR13 in the asymmetric unit of the crystal constituted an obstacle for the effective density improvement merely using the traditional methods. Therefore, a novel phase-extension method was developed in order to obtain interpretable high-resolution phases up to 2.3 Å. The flowchart of the current phase extension procedure is shown in Fig. 2A. An overview of the entire process is presented into the following steps:

**Figure 2:**
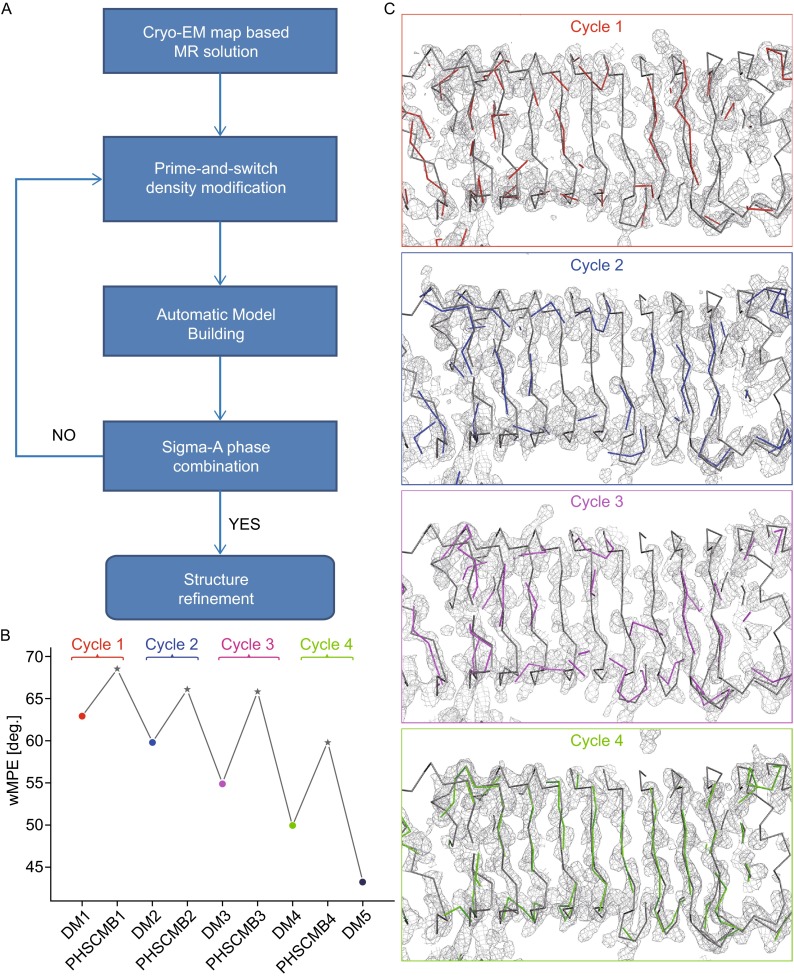
**An improved phase extension method for phasing crystal structures with low NCS using cryo-EM data**. (A) Flowchart of phase extension procedure. (B) Plot of figure-of-merit-weighted (FOM) mean phase errors (wMPE) calculated relative to the subsequently refined structure for the various steps of phase improvement. The cycle number of iteration in the flowchart of Fig. 2A is indicated above the plot with the various colors. DM, density modification in RESOLVE; PHSCMB, phase combination in SIGMAA. (C) A portion of the electron-density map for the various iterative phase-extension steps. The electron density maps are shown in light grey mesh representation at the various cycles, and the final refined model is supposed as grey ribbon onto the density. The ribbon representation of the models built automatically by RESOLVE is also shown with the color consistent with that used in Fig. 2B

1. The positioned EM map was used to calculate the phases for X-ray data up to the EM reconstruction resolution.
2. The phases were then improved with the prime-and-switch phasing method to remove model bias from the currently available phased map in RESOLVE (Terwilliger, 2000). (Density modification step, DM).
3. Automatic main-chain and side-chain model building was performed in the electron density map obtained in step 2 (Terwilliger, 2000). (Automatic model building step, AMB)
4. The phases calculated from auto-built models of step 3 were combined with the step-2 prime-and-switched phases in SIGMAA (Read, 1986) with the appropriate weights between the phases from the above two sources. (Phase combination step, PHSCMB)
5. Further prime-and-switch phase improvement was achieved by returning to step 2. The whole procedure can be performed iteratively, until the extended phases are good enough for map interpretation. The resulting electron density map was utilized for further automatic or manual model building, and final structure refinement.

In this case study, phases for X-ray data to 4.87 Å resolution were calculated for TLR13 based on the EM model and the MR positions, which gave map correlation coefficients relative to the final refined coordinates of 0.365 and 0.257 for main-chain and side-chain atoms, respectively (Fig. S1). A single run of traditional density modification procedure, including solvent flattening, histogram matching, and two-fold NCS averaging resulted in the phases with a figure-of-merit (FOM) weighted mean phase error of ~63° (Fig. 2B: Cycle 1). A portion of the electron-density map is shown in Fig. 2C: Cycle 1. It is difficult to trace the whole chain from this map. However, after automatic model building, RESOLVE could identify some correct residue positions, along with a number of wrong ones, which totally accounts for 38.56% of the candidate positions within 1.5 Å of a true Cα position (first row in Table S3). Combining the auto-built model phases with density-modified phases resulted in a sudden increase in the mean phase error to ~70°. Although the phase error temporarily rose up, this step could be considered akin to the temperature heating step in the simulated annealing method, which would help to escape from the local minimum to find the global minimum. Another cycle of density modification on the combined phases improved the resultant phases dramatically. The improved electron density map is shown in Fig. 2C: Cycle 2, and more correctly positioned Cα atoms were built automatically (Table S3). The whole procedure was repeated four times, alternating between density modification and phase combination. The mean phase error in the final cycle dropped to ~45° (Fig. 2B: Cycle 4), with a correlation coefficient of 0.77 (Fig. S1), which is sufficient for automatic model building and structure refinement.

In summary, based on our experience learned from the TLR13 structure, the process of the solution of the “phase problem” with high-resolution X-ray crystallographic data and a low-resolution cryo-EM reconstruction map may be divided into the following steps:

1. B-factor sharpened and FOM-weighted cryo-EM map should always be used as the starting point (Supplementary Materials).
2. The cryo-EM map is then corrected for the magnification error, according to the previous microscopic calibration (Supplementary Materials).
3. The cryo-EM reconstruction is placed at the center of a P1 cell with ‘crystal’ unit cell dimensions at least twice the model diameter. The structure factors can be generated by the inverse Fourier transform.
4. Molecular replacement is then performed to determine the orientation and position of the cryo-EM map in the crystal lattice.
5. Application of the phase extension procedure in this study is used to extend the phases to the highest resolution available from the X-ray data.
6. Final model building and structure refinement is performed.

## Footnotes

The research was funded by the National Basic Research Program (973 Program) (Nos. 2011CB911102 and 2015CB910104 to J.W.W., 2014CB910101 to J.C., 2010CB912401 and 2012CB917303 to H.W.W.). We acknowledge the China National Center for Protein Sciences Beijing for providing the facility support. We thank Professor Hai-Fu Fan, Professor Xinquan Wang, Dr. Elias Coutavas and Dr. Erik Debler for comments on the manuscript.

Jia Wang, Weiguang Wang, Wen Song, Zhifu Han, Heqiao Zhang, Jijie Chai, Hongwei Wang, and Jiawei Wang declare that they have no conflict of interest. This article does not contain any studies with human or animal subjects performed by the any of the authors.

## Electronic supplementary material

Supplementary material 1 (PDF 945 kb)
