# Supplementary material for: An improved method for phasing crystal structures with low non-crystallographic symmetry using cryo-electron microscopy data
Source: Protein Cell. 2015 Oct 27;6(12):919–23. doi: 10.1007/s13238-015-0219-4 (PMC4656207; doi:10.1007/s13238-015-0219-4)
Supplement: Supplementary file 1 — Supplementary material 1 (PDF 945 kb) [file 13238_2015_219_MOESM1_ESM.pdf]

Supplementary materials

This supplementary material file contains the following contents: Appendix, Materials and methods, Supplementary Figures (S1~S3) and Supplementary Tables (S1~S3).

Appendix

The described phase extension procedure is useful not only for EM reconstruction phasing, but also possibly for any low-resolution phase sources, *e.g.* a low-resolution X-ray density map cut-off (Abrescia et al., 2011). However, there are several special characteristic of cryo-EM reconstruction to affect the efficiency of the phase extension procedure, especially cryo-EM magnification, and EM map sharpening.

1. Effects of cryo-EM magnification on the phase extension

It is necessary to investigate how the cryo-EM magnification error takes effect on our phase extension algorithm, because this kind of error can be as large as 5% (Dodson, 2001; Rossmann et al., 2001; Xiong, 2008). Calibration of electron microscope magnification can be achieved by comparing the TLR13 cryo-EM map with the X-ray crystallographic map calculated from the final refined coordinates, because the energy bandwidth of the synchrotron X-ray source used in a crystallographic determination is usually known to within 1 part in 1000. The correlation coefficient between cryo-EM and X-ray map was calculated using EMfit (Rossmann et al., 2001), while varying the relative pixel size around the microscope-calibrated pixel size of 1.32 Å (Supplementary Fig. 2). The maximum correlation was reached at the pixel size of 1.307 Å. The initial EM map was then adjusted approximately to the correct pixel size. MR using the corrected EM

map as a search model was carried out using PHASER. A definite solution could be found with a Z-score of 12.7, lower than that of the unaltered EM map. However, the phase extension utilized only 4 cycles of iteration to reach the similar mean phase error, compared to 5 cycles with the original EM map. Therefore, the absolute value of translational functional Z-score between different search models may not be a good indicator in the EM magnification scale calibration. However, calibrated pixel size does provide more accurate initial phases during the phase extension.

2. Effects of cryo-EM map post-processing on phase extension

To observe molecular details at the resolution limit of the cryo-EM map, it is common to correct the structure factor amplitudes for the loss of contrast (Rosenthal and Henderson, 2003). Usually, both B-factor-sharpening and noise-weighting were applied to the structure factor amplitudes for contrast restoration. The sharpened map (left in Supplementary Fig. 3) shows more detailed molecular features than the unsharpened map (right in Supplementary Fig. 3). It is also important to investigate the effect of both B-factor-sharpening and FOM/noise weighting on phase extension procedure. A magnification-calibrated unsharpened and unweighted cryo-EM map was used as a model for structure solution by molecular replacement and phase extension. This map resulted in a PHASER Z-score of 12.9, a little higher than that of the sharpened one, and in a lower mean phase error after the first cycle of density modification. However, the mean phase errors of the sharpened map more quickly converged to the global minimum (Supplementary Fig. 3B, grey curve). Therefore, EM map post-processing, including B-

factor-sharpening and noise-weighting, seems to be a good choice for the cryo-EM map-based molecular replacement method.

In summary, the magnification error commonly encountered in electron microscopy didn’t much influence the orientation and translation determination by molecular replacement, if it is as large as 5%. However, the magnification error hindered the convergence tendency of phase extension. The more errors, the more cycles of phase extension are needed. B-factor-sharpening and FOM-weighting had similar effects on the phase extension convergence.

Materials and methods

Molecular replacement of cryo-EM map into the X-ray crystallographic lattice

To tackle the "phase problem" with crystallographic data, the reconstructed TLR13 dimer cryo-EM 3D density map was placed at the center of a large cubic box with the dimensions of 190.08 x 190.08 x 190.08 Å^3, and then inverse-Fourier-transformed into amplitudes and phases (Frigo and Johnson, 1998). The data were then used as a model for the molecular replacement search against the 2.3 Å X-ray data using PHASER (McCoy et al., 2007), which fortunately gave a definite solution with the translational function Z-score of 13.6.

Final model building and structural refinement

From the final phase-extended map, BUCCANEER (Cowtan, 2006) could build almost all the residues automatically, and additional missing residues in the auto-built model were added in COOT (Emsley and Cowtan, 2004; Emsley et al., 2010) manually. The structure was refined with PHENIX.refine (Adams et al., 2010; Adams et al., 2002) to R_{work} / R_{free} of 19.2% and 24.2%, respectively. The structural description of TLR13 will be presented elsewhere (Song et al., 2015).

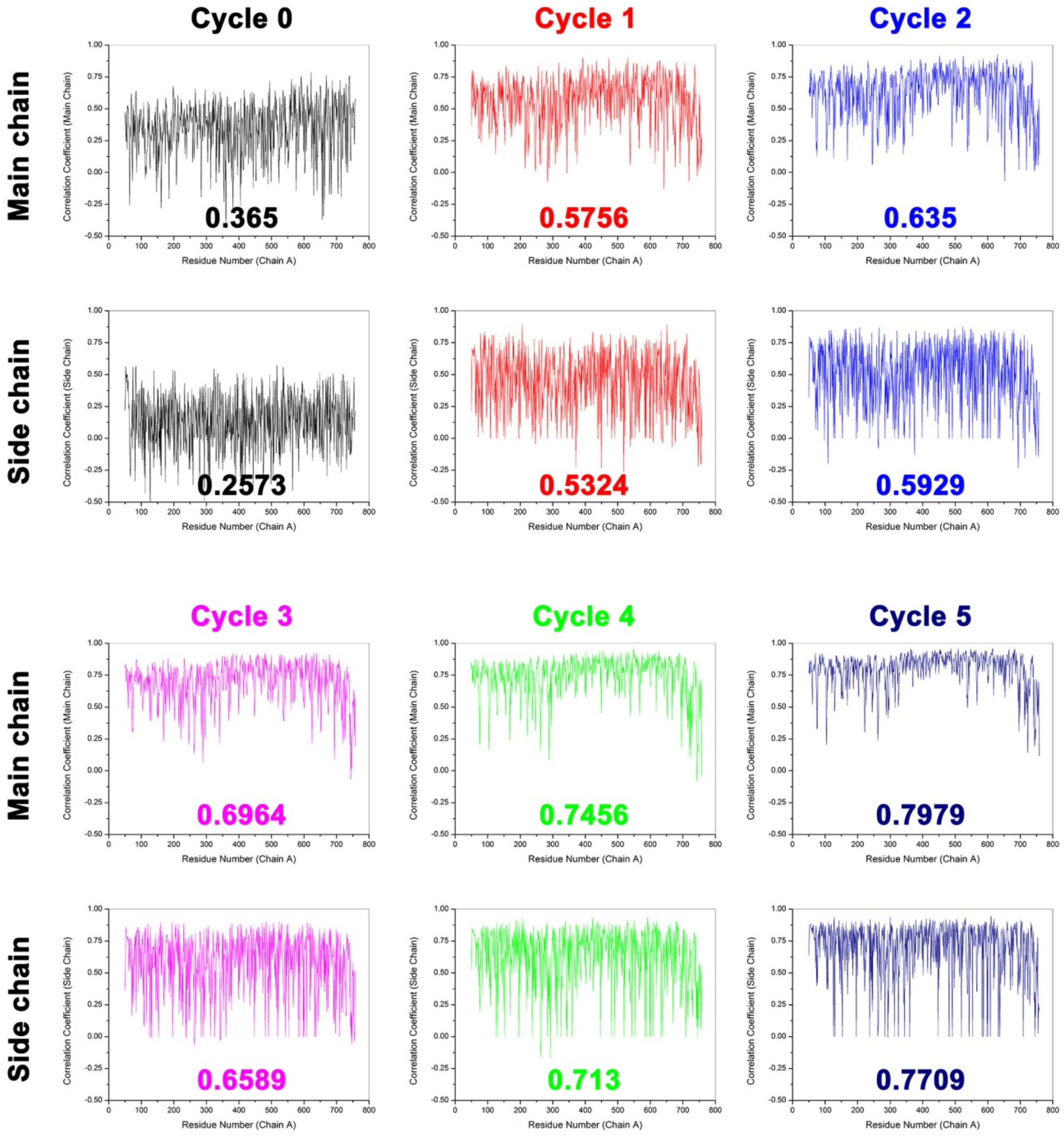

Supplementary Figure 1 The residue-by-residue correlation coefficient (CC) between the electron density map concerned and the final refined structure. CC for main chain atoms is shown on the top row, and side chain on the bottom row for six different states. The overall CC is shown just on top of the line plot.

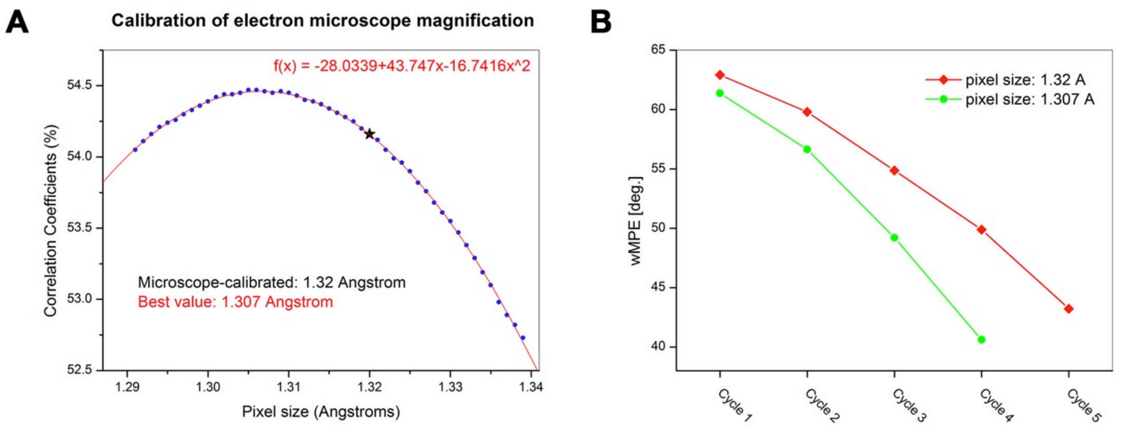

Supplementary Figure 2 Determination of EM magnification scale and its effects on the phase extension. (A) correlation coefficients between the TLR13 cryo-EM map and the map based on crystallographic model coordinates were utilized to calibrate the magnification scale. The microscopy calibrated value was reported to 1.32 Å. However, after parabola fitting the CC vs pixel size curve, the maximum appeared at 1.307 Å. (B) magnification scale calibrated cryo-EM map was used for the molecular replacement and phase extension. Calibrated map resulted in the a little bit lower TFZ than that uncalibrated. However, the overall number of cycles of phase extension was decreased, also for the final phase error.

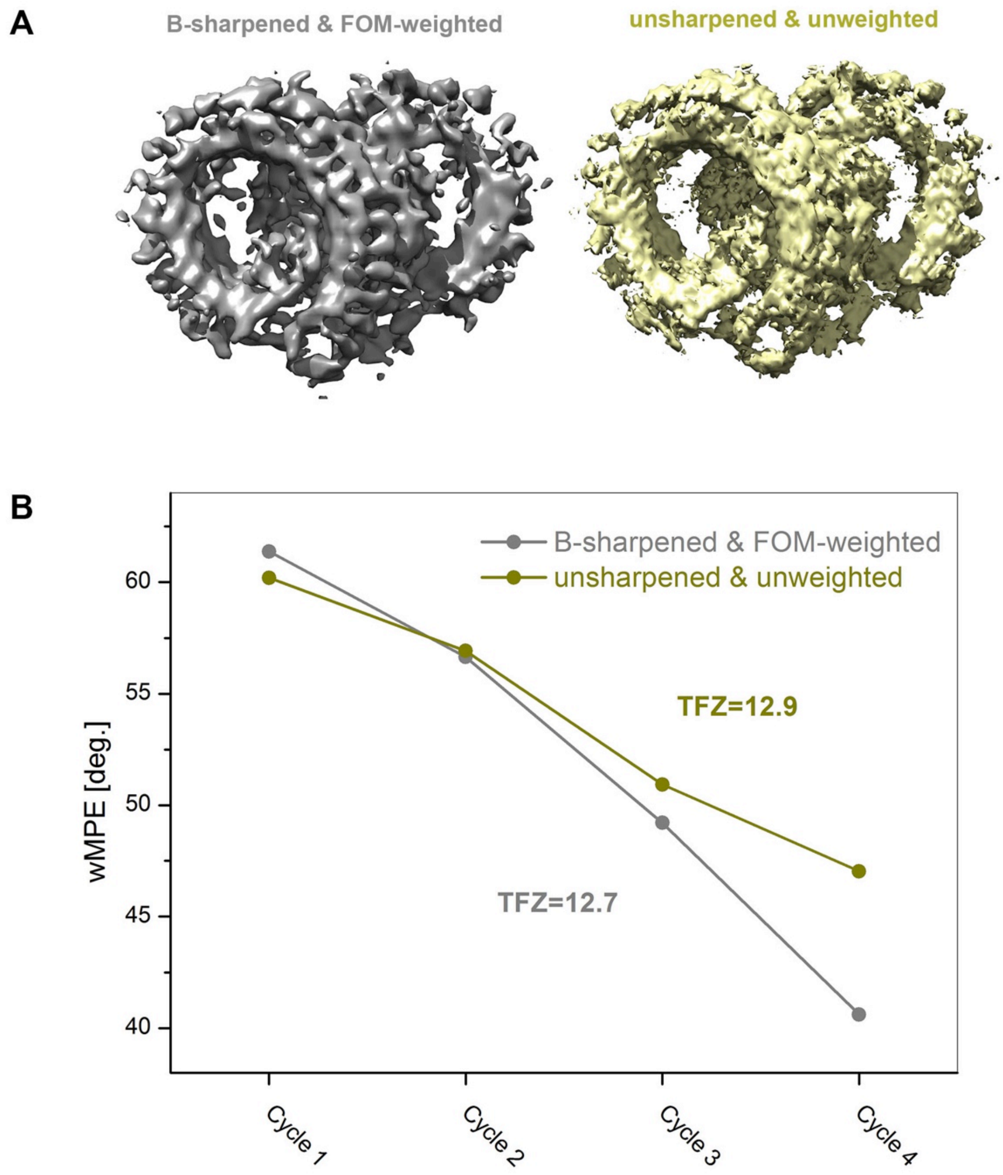

Supplementary Figure 3 B-factor sharpening and FOM weighting of cryo-EM map on the phase extension. (A) B-sharpened and FOM-weighted cryo-EM map was shown on the left, unaltered on the right. (B) mean phase error effects for the sharpened and unprocessed cryo-EM on the phase extension procedure. Both of the cryo-EM maps have been calibrated for the magnification scale according to Supplementary Fig. 2.

Supplementary Table 1 X-ray data-collection parameters and statistics

Wavelength (Å)	1.0
CCD detector	ADSC Q315 CCD
Resolution range (Å)	50.0-2.30 (2.54-2.30)
Space group	$P2_12_12_1$
Unit-cell parameters (Å,°)	112.55, 115.21, 167.61, 90, 90, 90
Multiplicity	8.5 (6.4)
Completeness (%)	92.9 (60.3)
$\langle I\rangle/\langle\sigma(I)\rangle$	27.5 (3.1)
R_{merge} (%)	7.8 (49.6)

Values in parentheses are for the high-resolution bin. Based on Song et al. (2015)

Supplementary Table 2 Cryo-EM image collection and processing

No. of particles picked	~262,000
Reconstruction package	Relion 1.3 (Scheres, 2012)
Before C2 symmetry constraints applied	
Resolution (gold-standard FSC at 0.143)	5.76 Å
B-factor	270 $Å^2$
After C2 symmetry constraints applied	
Resolution (gold-standard FSC at 0.143)	4.87 Å
B-factor	230 $Å^2$

Based on Song et al. (2015)

Supplementary Table 3 Completeness and accuracy of the resulting chain trace by RESOLVE at the different cycle of iterations.

No. of cycle	Completeness (%)	Accuracy (%)
1	38.56	79.94
2	39.55	80.92
3	40.47	79.25
4	46.47	86.35

References:

Abrescia, N.G., Grimes, J.M., Oksanen, H.M., Bamford, J.K., Bamford, D.H., and Stuart, D.I. (2011). The use of low-resolution phasing followed by phase extension from 7.6 to 2.5 A resolution with noncrystallographic symmetry to solve the structure of a bacteriophage capsid protein. Acta crystallographica Section D, Biological crystallography 67, 228-232.

Adams, P.D., Afonine, P.V., Bunkóczi, G., Chen, V.B., Davis, I.W., Echols, N., Headd, J.J., Hung, L.-W., Kapral, G.J., and Grosse-Kunstleve, R.W. (2010). PHENIX: a comprehensive Python-based system for macromolecular structure solution. Acta Crystallographica Section D: Biological Crystallography 66, 213-221.

Adams, P.D., Grosse-Kunstleve, R.W., Hung, L.-W., Ioerger, T.R., McCoy, A.J., Moriarty, N.W., Read, R.J., Sacchettini, J.C., Sauter, N.K., and Terwilliger, T.C. (2002). PHENIX: building new software for automated crystallographic structure determination. Acta Crystallographica Section D: Biological Crystallography 58, 1948-1954.

Cowtan, K. (2006). The Buccaneer software for automated model building. 1. Tracing protein chains. Acta Crystallographica Section D: Biological Crystallography 62, 1002-1011.

Dodson, E.J. (2001). Using electron-microscopy images as a model for molecular replacement. Acta crystallographica Section D, Biological crystallography 57, 1405-1409.

Emsley, P., and Cowtan, K. (2004). Coot: model-building tools for molecular graphics. Acta Crystallographica Section D: Biological Crystallography 60, 2126-2132.

Emsley, P., Lohkamp, B., Scott, W., and Cowtan, K. (2010). Features and development of Coot. Acta Crystallographica Section D: Biological Crystallography 66, 486-501.

Frigo, M., and Johnson, S.G. (1998). FFTW: An adaptive software architecture for the FFT. Paper presented at: Acoustics, Speech and Signal Processing, 1998 Proceedings of the 1998 IEEE International Conference on (IEEE).

McCoy, A.J., Grosse-Kunstleve, R.W., Adams, P.D., Winn, M.D., Storoni, L.C., and Read, R.J. (2007). Phaser crystallographic software. Journal of applied crystallography 40, 658-674.

Rosenthal, P.B., and Henderson, R. (2003). Optimal determination of particle orientation, absolute hand, and contrast loss in single-particle electron cryomicroscopy. Journal of molecular biology 333, 721-745.

Rossmann, M.G., Bernal, R., and Pletnev, S.V. (2001). Combining electron microscopic with X-ray crystallographic structures. Journal of Structural Biology 136, 190-200.

Scheres, S.H. (2012). RELION: implementation of a Bayesian approach to cryo-EM structure determination. Journal of Structural Biology 180, 519-530.

Song, W., Wang, J., Han, Z., Zhang, Y., Zhang, H., Wang, W., Chang, J., Xia, B., Fan, S., Zhang, D., *et al.* (2015). Structural basis for specific recognition of single-stranded RNA by toll-like receptor 13. Nature structural & molecular biology.

Xiong, Y. (2008). From electron microscopy to X-ray crystallography: molecular-replacement case studies. Acta crystallographica Section D, Biological crystallography 64, 76-82.
